# Analysis of clinical characteristics and predictive model for effective treatment of tinnitus in patients with transient compound sound therapy

**DOI:** 10.3389/fneur.2024.1515953

**Published:** 2024-12-09

**Authors:** Hao Yuan, Peng-Wei Ma, Jia-Wei Chen, Wei-Long Wang, Wei Gao, Pei-Heng Lu, Xue-Rui Ding, Yu-Qiang Lun, Zi Wang, Lian-Jun Lu

**Affiliations:** Department of Otolaryngology Head and Neck Surgery, Tangdu Hospital, Air Force Medical University, Xi’an, China

**Keywords:** tinnitus, sound therapy, prognosis, outcome prediction, nomogram

## Abstract

**Purpose:**

This study explored the clinical characteristics of patients with tinnitus who responded to sound therapy and established a predictive model to evaluate the effectiveness of this therapy according to the clinical characteristics.

**Methods:**

A retrospective analysis was performed on 991 subjective tinnitus patients who received compound sound therapy in the Department of Otolaryngology of the local hospital from November 2019 to January 2022.

**Results:**

We found that tinnitus patients with different therapeutic effects had significant differences in the tinnitus side (*p* = 0.007), tone loudness distortion feedback test (FBT) (*p* = 0.000), residual inhibition test (RIT) (*p* = 0.000), tinnitus frequency (*p* = 0.012) and sensation level (*p* = 0.023). The corresponding variables were screened by univariate logistic regression, and the selected variables were analyzed using multivariate logistic regression. The results showed that FBT (*p* = 0.003), RIT (*p* = 0.000) and tinnitus frequency (*p* = 0.029) were independent risk factors affecting the efficacy of compound sound therapy. A predictive model and nomogram for the efficacy of compound sound therapy for tinnitus were constructed based on independent risk factors. The area under the curve (AUC) of the model constructed in this study was 0.766 (95% CI = 0.725–0.807), indicating a certain prediction ability. The calibration curve revealed that the predicted results were in good agreement with the actual results.

**Conclusion:**

The model can predict the prognosis of tinnitus in patients receiving compound sound therapy and help otolaryngologists make the best clinical decisions regarding tinnitus treatment.

## Introduction

Tinnitus is the conscious perception of auditory sensations in the absence of corresponding external stimuli and is mainly divided into subjective tinnitus and objective tinnitus (1). Tinnitus can impair speech comprehension, making it difficult for those who experience it to concentrate, interfering with nighttime rest and increasing anxiety and depression (2). Tinnitus affects more than 740 million adults globally and is perceived as a major problem by more than 120 million people, mostly those aged 65 years or older (3).

The pathogenesis and pathophysiological pathways (4) of tinnitus development are unclear. Multiple forms of tinnitus reflect complex interactions between peripheral and central mechanisms in the auditory pathway (5). Previous studies have suggested that hearing loss leads to reduced upload of auditory impulses, resulting in reduced neuron impulses in the brain region of the corresponding frequency in the auditory cortex of the brain, plasticity changes in the auditory cortex, increased excitability of the auditory cortex surrounding the frequency, and an increased spontaneous discharge rate of neurons, leading to tinnitus (6). Recent studies on rodents and humans have found that emotional and cognitive transmission in the brain such as temporal, parietal, sensorimotor, and limbic cortex are involved in the occurrence of tinnitus (7, 8).

Many treatments are available for tinnitus; however, their efficacy is unsatisfactory due to individual differences, and some treatments are only effective for some patients. According to the tinnitus pathogenesis hypothesis, attempts to restore sound input from the cochlea to the cerebral cortex may reduce tinnitus symptoms, thus, some researchers use hearing aids or tinnitus masking devices to reduce tinnitus symptoms (9). Others have attempted to use oral drugs (10), repetitive transcranial magnetic stimulation (11), transcranial direct current stimulation (12, 13), and vagus nerve stimulation (14) to treat tinnitus; however, the effectiveness of these treatments is not good. The efficacy of cognitive behavioral therapy, a psychological intervention rooted in the cognitive and behavioral traditions of psychology, has been empirically demonstrated in enhancing tinnitus patients’ quality of life and alleviating distress associated with tinnitus (15, 16). However, due to limited accessibility, cognitive behavioral therapy is not widely utilized by medical professionals for the treatment of tinnitus.

Because of the difference in the treatment effect among tinnitus patients, customized treatment of tinnitus may be a key future research direction. Stracke et al. administered compound sound therapy to tinnitus patients for 12 months and found that subjective tinnitus loudness and negative emotion related to tinnitus in patients in the experimental group were significantly reduced, while the corresponding indicators in the placebo group and the blank control group did not change (17). Okamoto et al. found that patients’ tinnitus loudness could be significantly reduced by the combination of sound therapy (18). However, Liu et al. found in a large-sample, single-arm study that the reduction in the Tinnitus Handicap Inventory (THI) score after receiving customized music therapy depended on the severity of the patient’s tinnitus (19).

The effect of compound sound therapy on tinnitus patients is uncertain. Therefore, exploring the clinical characteristics of effective compound sound therapy in tinnitus patients and establishing a model to predict the effectiveness of this therapy according to the clinical characteristics are substantially significant, which will improve its effectiveness in tinnitus patients.

## Materials and methods

### Patients

**Figure 1:**
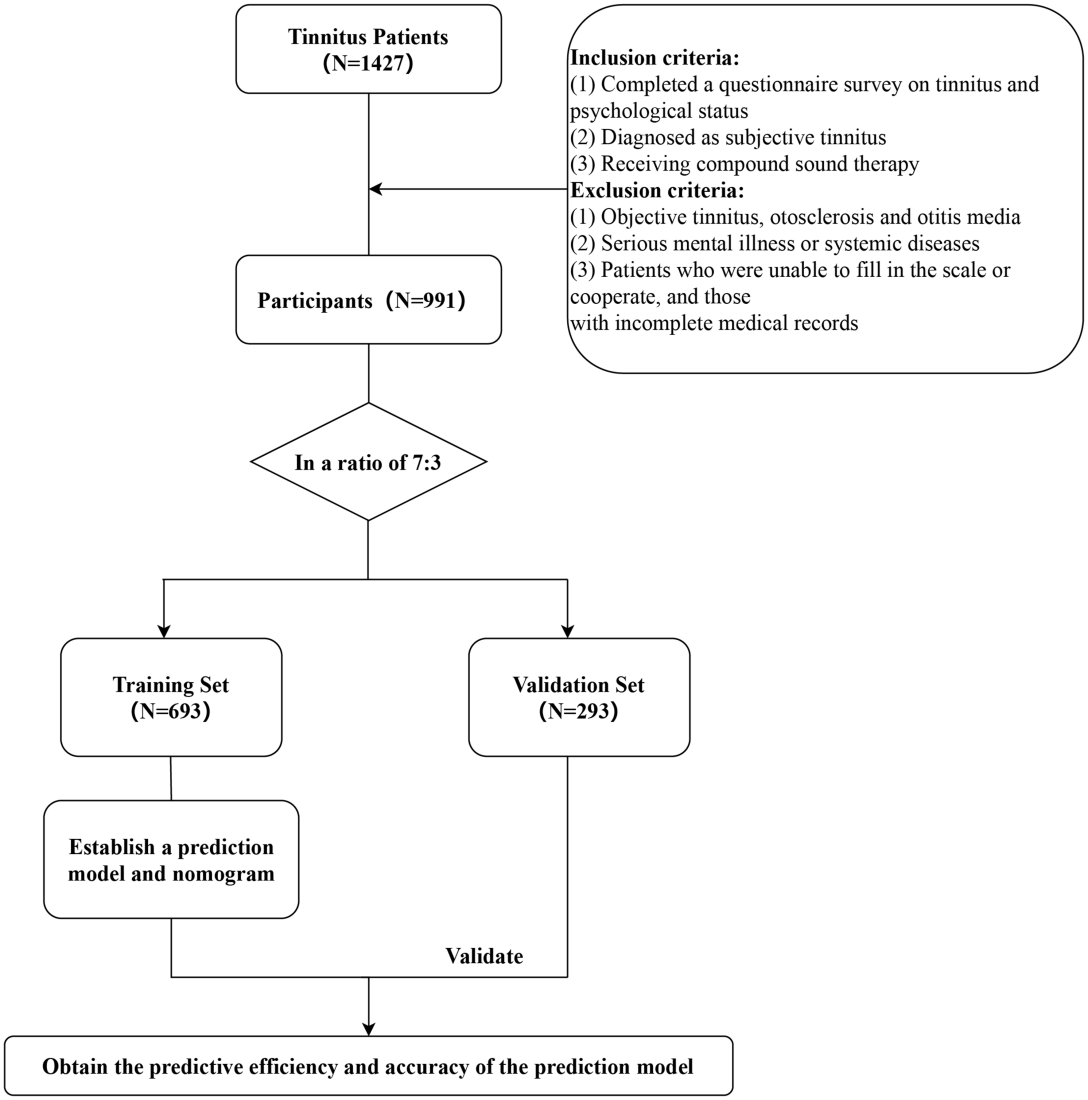
Flow chart.

This retrospective study was conducted at the Hearing Center of the Department of Otolaryngology at the local hospital. The study included 991 patients with subjective tinnitus who received short-term compound sound therapy at the Department of Otolaryngology at the local hospital from November 2019 to January 2022. All patients completed a questionnaire survey on tinnitus and their psychological status, underwent numerous audiological examinations, and were finally diagnosed with subjective tinnitus. Patients with various other ear diseases such as objective tinnitus, otosclerosis and otitis media were excluded. Patients with serious mental illness or systemic diseases such as cardiovascular and cerebrovascular diseases, those who were unable to complete the scale or cooperate, and those with incomplete medical records were excluded. The investigation was conducted in accordance with the tenets of the Declaration of Helsinki, as depicted in Figure 1, and approved by the Medical Ethics Committee of the local hospital.

### Precision tinnitus testing

The patients underwent pure tone audiometry in acoustically treated compartments, recording auditory thresholds of 125 Hz, 250 Hz, 500 Hz, 1,000 Hz, 2000 Hz, 4,000 Hz, and 8,000 Hz. The average hearing threshold of all patients was calculated as the average hearing thresholds of 500 Hz, 1,000 Hz, and 2000 Hz. In addition, all tinnitus patients were matched with fine-tuned tinnitus of a 1/12x frequency according to audiogram data, and tinnitus-related data were recorded. These data included the tinnitus type, side, loudness and frequency.

All patients underwent the tone loudness distortion feedback test (FBT) and residual inhibition test (RIT). The FBT is used to provide a specific sound stimulus to the ear with tinnitus according to the loudness of the tinnitus, and after stimulation for a few seconds, the patient is asked whether the tinnitus has changed under the pure tone stimulus. The results were categorized as follows: 1, completely positive (tinnitus was not audible); 2, partial positive (tinnitus became quieter); 3, negative (no change in tinnitus perception); and 4, rebound effect (tinnitus became louder). RIT is a 10 dB narrow band noise stimulus that is administered to the patient over the tinnitus loudness threshold for 1 min, and the patient is asked whether the tinnitus changes after the stimulus sound stops. The results were categorized as follows: 1, completely positive (tinnitus was not audible); 2, partial positive (tinnitus became quieter); 3, negative (no change at tinnitus perception); and 4, rebound effect (tinnitus became louder).

The tinnitus loudness was subtracted from the tinnitus frequency hearing threshold, and this difference was defined as the tinnitus loudness perception level. The tinnitus type, loudness, frequency, FBT and RIT were selected as the characteristics of patients with poor hearing.

### Tinnitus severity assessment

Patients completed two questionnaires to measure tinnitus severity. The THI, which includes functional, emotional and severity-related questions, was used to comprehensively assess each patient’s status (20). Each question on the scale had 3 answer options, in which the answer “yes” was worth 4 points, “sometimes” was worth 2 points, and “no” was worth 0 points. According to the difference in the total score, the patients were categorized as Grade 1 (slight, THI total score of 0–16 points), Grade 2 (mild, THI total score of 18–36), Grade 3 (moderate, THI total score 38–56), Grade 4 (severe, THI total score of 58–76), and Grade 5 (catastrophic, THI total score of 78–100). De Ridder and his colleagues proposed the concept of “Tinnitus Disorder” which reflects the auditory component and the associated suffering (21). We combined severe and catastrophic tinnitus severity into one group, where the lives of tinnitus patients were greatly affected by tinnitus. We combined tinnitus patients with slight, mild, and moderate tinnitus severity into one group, where the lives of tinnitus patients were less affected by tinnitus. The visual analog scale (VAS) is a clinical method used to assess the intensity of pain on a scale from 0 to 10, with “0” indicating no pain and “10” representing unendurable and severe pain. Clinically, “0” is “painless, “1–3″ is “mild pain, “4–6″ is “moderate pain, and “7–10″ is severe pain (22).

### Treatment protocol

The prescription for compound sound therapy consists of 3 parts. The first part is matched according to the patient’s tinnitus frequency, mainly using narrowband noise. The second part is for different natural sounds, such as rain and bird calls, which can be divided into low-frequency, medium-frequency, high-frequency and multi-frequency sounds, and the masking frequency of natural sounds is selected according to the frequency of the patient’s tinnitus site. Part 3 involves the frequency of music material according to the patient’s personal preference. All three pieces of music must effectively cover the tinnitus sound, and the loudness of the synthesized music file is approximately 10–15 dB above the tinnitus loudness threshold. The duration of sound therapy should be 10–15 min. Sound therapy is considered effective if the patient’s conscious tinnitus lessens or disappears after receiving sound therapy for 10–15 min and ineffective if they experience no change or aggravated tinnitus symptoms.

### Statistical analysis

SPSS 26.0 (IBM, Armonk, NY, USA) and Graphpad 8.0 (Insightful Science, USA) were used for the statistical analyses and the production of the data. Normally distributed continuous variables are represented as means ± standard deviations, while non-normally distributed data are described as medians (interquartile ranges). Statistical comparisons between groups were performed using analysis of variance or the Mann–Whitney test for continuous variables. Categorical variables were compared using the χ^2^ test or Fisher’s exact probability test. The demographic characteristics, acoustic characteristics, severity of tinnitus and impact degree of tinnitus in patients with different sound treatment effects were compared.

Patients were randomly divided into a training set and a validation set at a 7:3 ratio. The demographic characteristics, acoustic characteristics and severity of tinnitus were compared between the training set and the validation set. In the training set, the demographic characteristics, acoustic characteristics of tinnitus, tinnitus severity and treatment efficacy were analyzed using univariate logistic regression. Factors with *p* < 0.05 in the univariate analysis were included in the multivariate logistic regression analysis. The model was established by the forward method in logistic regression, with *α* in = 0.05 and α out = 0.10. Based on the results of the multiple logistic regression analysis, columns were drawn using the RMS package version R3.0.^1^ The nomogram was constructed by scaling each regression coefficient in the multivariate logistic regression from 0 to 100 points. The variable with the highest beta coefficient (absolute value) was scaled to 100 points to represent its impact. These points were added across the independent variables to reach the total point, which was converted into a predicted probability. A corresponding ROC curve was generated to measure the predictive performance of the nomogram through the calibration curve of 1,000 bootstrap samples to reduce overfitting bias. The efficiency of the nomogram was then evaluated using a validation set, and the corresponding ROC curve and calibration curve of 1,000 bootstrap samples were obtained. *p* < 0.05 was considered to indicate statistical significance.

## Results

### Comparison of the characteristics of tinnitus patients with different treatment efficacies of sound therapy

This study included patients with 991 subjective tinnitus treated at the Department of Otolaryngology local hospital from November 2019 to January 2022 who received transient compound sound therapy. Treatment was effective for 752 patients and ineffective for 239 patients. Patients with subjective tinnitus who experienced different therapeutic effects exhibited significant differences in the tinnitus location (*p* = 0.007), FBT (*p* = 0.000), RIT (*p* = 0.000), tinnitus frequency (*p* = 0.012) and sensation level (*p* = 0.023) (Table 1). No significant differences in sex (*p* = 0.737), age (*p* = 0.126), type of tinnitus (*p* = 0.585), type of tinnitus sounds (*p* = 0.680), degree of hearing impairment (*p* = 0.566), THI score (*p* = 0.050), VAS score (*p* = 0.263), impact degree (*p* = 0.216) or average hearing threshold (*p* = 0.181) were observed (Table 1).

**Table 1:** Comparison of characteristics of tinnitus patients with different responses to sound therapy.

Variable	Ineffective (*N* = 239)	Effective (*N* = 752)	*P* value	Test value
Sex				
Female	107 (44.80%)	346 (46.00%)	0.737	0.113
Male	132 (55.20%)	406 (54.00%)		
Age, years				
0–17	5 (2.10%)	8 (1.10%)	0.126	5.714
18–44	112 (46.90%)	335 (44.50%)		
45–65	107 (44.80%)	327 (43.50%)		
>65	15 (6.30%)	82 (10.90%)		
Tinnitus location				
Left	83 (34.70%)	215 (28.60%)	0.007	10.013
Right	72 (30.10%)	185 (24.60%)		
Bilateral	84 (35.10%)	352 (46.80%)		
Types of tinnitus				
Acute	86 (36.00%)	275 (36.60%)	0.585	1.074
Subacute	23 (9.60%)	89 (11.80%)		
Chronic	130 (54.50%)	388 (51.60%)		
Types of tinnitus sounds				
Persistent	182 (76.20%)	573 (76.20%)	0.680	1.508
Intermittent	13 (5.40%)	56 (7.40%)		
Obvious in quiet environments	43 (18.00%)	120 (16.00%)		
Obvious in noisy environments	1 (0.40%)	3 (0.40%)		
Degree of hearing impairment				
0–25 dB	131 (54.80%)	444 (59.00%)	0.566	3.889
26–40 dB	47 (19.70%)	132 (17.60%)		
41–55 dB	29 (12.10%)	99 (13.20%)		
56–70 dB	16 (6.70%)	46 (6.10%)		
71–90 dB	12 (5.00%)	22 (2.90%)		
>91 dB	4 (1.70%)	9 (1.20%)		
FBT				
Negative	16 (6.70%)	16 (2.10%)	0.000	65.401
Partially positive	106 (44.40%)	163 (21.70%)		
Completely positive	117 (49.00%)	573 (76.20%)		
RIT				
Rebound	1 (0.40%)	0 (0.00%)	0.000	147.392
Negative	97 (40.60%)	71 (9.40%)		
Partially positive	119 (49.80%)	429 (57.00%)		
Completely positive	22 (9.20%)	252 (33.50%)		
Tinnitus frequency				
(0, 500 Hz]	79 (33.10%)	185 (24.60%)	0.012	8.864
(500,3,000 Hz]	23 (9.60%)	57 (7.60%)		
(3,000,8,000 Hz]	137 (57.30%)	510 (67.80%)		
THI				
Slight	43 (18.00%)	91 (12.10%)	0.05	9.507
Mild	62 (25.90%)	224 (29.80%)		
Moderate	53 (22.20%)	214 (28.50%)		
Severe	52 (21.80%)	137 (18.20%)		
Catastrophic	29 (12.10%)	86 (11.40%)		
VAS				
Mild	126 (52.70%)	383 (50.90%)	0.263	2.670
Moderate	106 (44.40%)	358 (47.60%)		
Severe	7 (2.90%)	11 (1.50%)		
Impact degree				
Mild	158 (66.10%)	529 (70.30%)	0.219	1.531
Severe	81 (33.90%)	223 (29.70%)		
Sensation level, dB	6 (6)	5 (6)	0.023	−2.275
Average hearing threshold, dB	12 (28)	20 (26)	0.181	−1.337

### Univariate and multivariate analyses

In this study, all patients with subjective tinnitus who received compound sound therapy were divided into a training set and a verification set at a ratio of 7:3. The training set included 693 patients with subjective tinnitus and the validation set included 298 patients. Table 2 lists the clinical characteristics of patients with subjective tinnitus in the training and validation sets, which were similar. A total of 526 (75.90%) and 226 (75.80%) patients with subjective tinnitus were included in the two datasets, respectively.

**Table 2:** Comparison of characteristics of tinnitus patients in different datasets (training set vs. validation set).

Variable	Training set (*N* = 693)	Validation set (*N* = 298)	*p* value	Test value
Treatment effectiveness				
Ineffective	167 (24.10%)	72 (24.20%)	0.983	0.000
Effective	526 (75.90%)	226 (75.80%)		
Sex				
Female	309 (44.60%)	144 (48.30%)	0.279	1.170
Male	384 (55.40%)	154 (51.70%)		
Age, years				
0–17	8 (1.20%)	5 (1.70%)	0.563	2.046
18–44	321 (46.30%)	126 (42.30%)		
45–65	295 (42.60%)	139 (46.60%)		
>65	69 (10.00%)	28 (9.40%)		
Tinnitus location				
Left	218 (31.50%)	80 (26.80%)	0.301	2.400
Right	173 (25.00%)	84 (28.20%)		
Bilateral	302 (43.60%)	134 (45.00%)		
Types of tinnitus				
Acute	251 (36.20%)	110 (36.90%)	0.146	3.843
Subacute	70 (10.10%)	42 (14.10%)		
Chronic	372 (53.70%)	146 (49.00%)		
Types of tinnitus sounds				
Persistent	529 (76.30%)	226 (75.80%)	0.680	1.508
Intermittent	45 (6.50%)	24 (8.10%)		
Obvious in quiet environments	115 (16.60%)	48 (16.10%)		
Obvious in noisy environments	4 (0.60%)	0 (0.00%)		
Degree of hearing impairment				
0–25 dB	405 (58.40%)	170 (57.00%)	0.056	10.776
26–40 dB	119 (17.20%)	60 (20.10%)		
41–55 dB	99 (14.30%)	29 (9.70%)		
56–70 dB	42 (6.10%)	20 (6.70%)		
71–90 dB	23 (3.30%)	11 (3.70%)		
>91 dB	5 (0.70%)	8 (2.70%)		
FBT				
Negative	20 (2.90%)	12 (4.00%)	0.138	3.963
Partially positive	200 (28.90%)	69 (23.20%)		
Completely positive	473 (68.30%)	217 (72.80%)		
RIT				
Rebound	0 (0.00%)	1 (0.30%)	0.275	3.879
Negative	121 (17.50%)	47 (15.80%)		
Partially positive	388 (56.00%)	160 (53.70%)		
Completely positive	184 (26.60%)	90 (30.20%)		
Tinnitus frequency				
(0, 500 Hz]	190 (27.40%)	74 (24.80%)	0.068	5.37
(500,3,000 Hz]	47 (6.80%)	33 (11.10%)		
(3,000,8,000 Hz]	456 (65.80%)	191 (64.10)		
THI				
Slight	96 (13.90%)	38 (12.80%)	0.966	0.569
Mild	201 (29.00%)	85 (28.50%)		
Moderate	188 (27.10%)	79 (26.50%)		
Severe	129 (18.60%)	60 (20.10%)		
Catastrophic	79 (11.40%)	36 (12.10%)		
VAS				
Mild	366 (52.80%)	143 (48.00%)	0.376	1.956
Moderate	315 (45.50%)	149 (50.00%)		
Severe	12 (1.70%)	6 (2.00%)		
Impact degree				
Mild	485 (70.00%)	202 (67.80%)	0.491	0.474
Severe	208 (30.00%)	96 (32.20%)		
Sensation level, dB	5 (6)	5 (7)	0.232	−1.196
Average hearing threshold, dB	22 (24)	21 (27)	0.617	−0.5

The univariate logistic regression results for the training set are shown in Table 3. The tinnitus location (*p* = 0.037), FBT (*p* = 0.000), RIT (*p* = 0.000), tinnitus frequency (*p* = 0.000), and average hearing threshold (*p* = 0.039) were included in the multivariate logistic regression analysis to identify independent risk factors affecting treatment efficacy.

**Table 3:** Univariate logistic regression analysis of the treatment effectiveness.

Variable	OR (95% CI)	*p* value
Sex	1.119 (0.789–1.587)	0.527
Age	1.243 (0.956–1.617)	0.104
Tinnitus location	1.241 (1.014–1.519)	0.037
Tinnitus type	0.974 (0.808–1.175)	0.787
Types of tinnitus sounds	0.977 (0.783–1.219)	0.834
Hearing impairment degree	0.875 (0.758–1.009)	0.067
FBT	2.649 (1.939–3.620)	0.000
RIT	4.704 (3.393–6.521)	0.000
Tinnitus frequency	1.400 (1.159–1.691)	0.000
THI	0.960 (0.832–1.109)	0.580
VAS	0.913 (0.659–1.263)	0.582
Impact degree	0.723 (0.500–1.047)	0.086
Sensation level	0.990 (0.958–1.023)	0.557
Average hearing threshold	0.991 (0.983–1.000)	0.039

Multivariate logistic regression analysis revealed that FBT (OR = 1.688, *p* = 0.003), RIT (OR = 3.997, *p* = 0.000) and tinnitus frequency (OR = 1.263, *p* = 0.029) were independent risk factors for combined sound therapy.

### Development and validation of a therapeutic effectiveness nomogram

**Figure 2:**
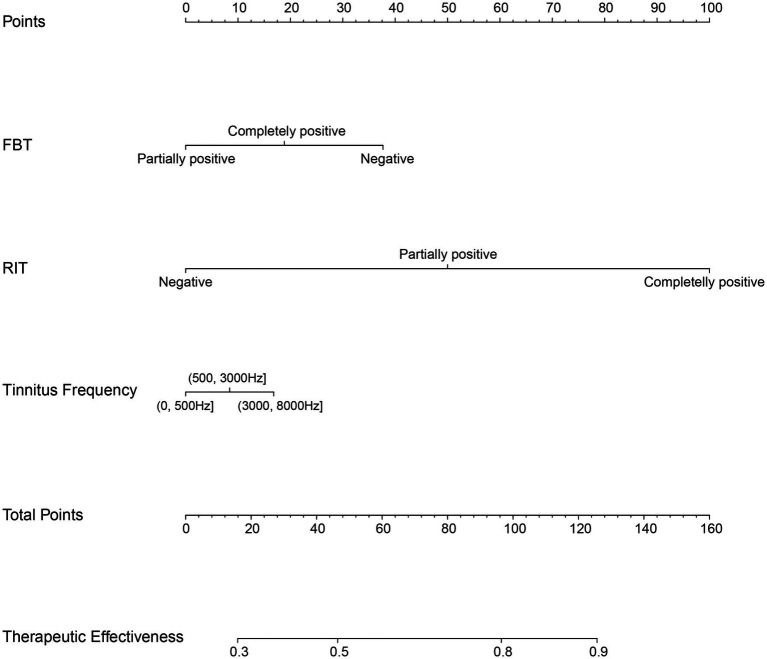
Nomogram for predicting the therapeutic effectiveness of customized compound sound therapy in patients with subjective tinnitus. For example: After examination, a tinnitus patient was found to have a negative FBT and a partially positive RIT, with tinnitus frequency between 3,000–8,000 Hz. The patient’s corresponding total points is 125. The nomogram predicts that the effectiveness of compound sound therapy for treating tinnitus is approximately 90%.

**Figure 3:**
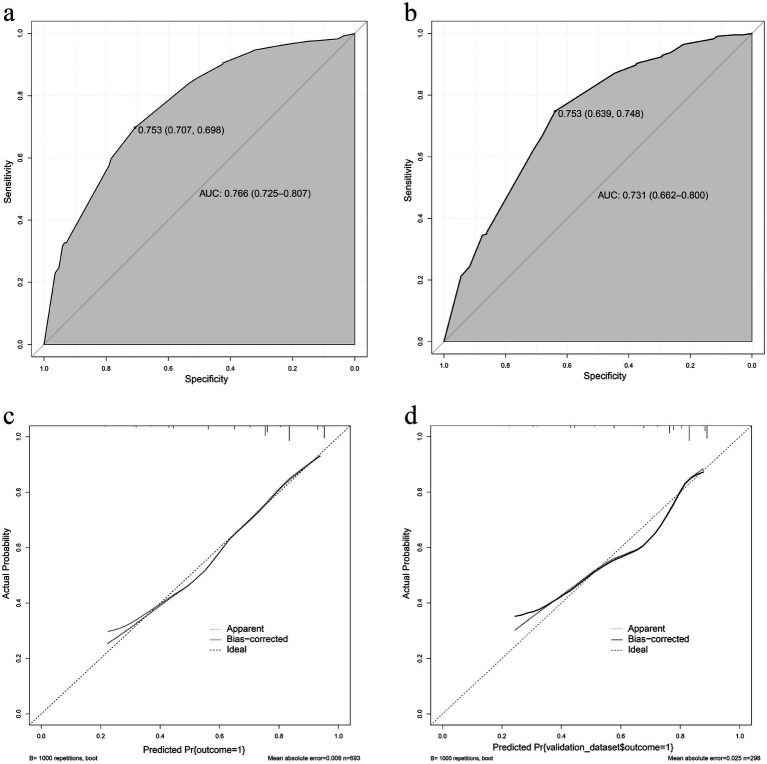
**(A)** The receiver operating characteristic curve of the nomogram in the training set. **(B)** The receiver operating characteristic curve of the nomogram in the validation set. **(C)** The effectiveness of the nomogram in estimating the predictive performance of compound sound therapy on subjective tinnitus in the training set (*n* = 693). **(D)** The effectiveness of the nomogram in estimating the predictive performance of compound sound therapy on subjective tinnitus in the validation set (*n* = 298).

The effectiveness of compound sound therapy for patients with subjective tinnitus based on the results of the multiple logistic regression analysis of the training set (Figure 2). Each predictor’s individual scores in the column chart were summed to obtain a total score and used to calculate the probability that the corresponding compound sound treatment would be effective against subjective tinnitus. This approach helps to evaluate the efficacy of compound sound therapy for subjective tinnitus. Internal validation of the model built with the training set using bootstrapping showed that the nomogram had good accuracy in predicting the effectiveness of compound sound therapy for patients with subjective tinnitus. The AUC was 0.766 (95% CI = 0.725–0.807) (Figure 3A). In addition, the calibration curve of the nomogram showed that the actual diagnosis was in good agreement with the predicted probability (Figure 3C).

In the validation set, the AUC of the nomogram for predicting the effectiveness of compound sound therapy for subjective tinnitus was 0.731 (95% CI = 0.662 to 0.800) (Figure 3B). A good calibration curve was generated for the risk estimation (Figure 3D).

## Discussion

Researchers have tried various methods to treat or even cure tinnitus, however, to date, no single treatment has provided satisfactory results. The effectiveness of sound therapy in altering tinnitus perception has been recognized for centuries, and in recent decades, the use of sound or sound enrichment to mask or suppress tinnitus or disrupt the neural activity that causes tinnitus has become a central part of clinical management (23). At present, the pathogenesis of tinnitus remains unclear (24). Therefore, most treatments focus on alleviating the associated distress caused by tinnitus, and treatments that directly eliminate tinnitus are lacking. Sound therapy is a widely used tinnitus treatment because it is noninvasive, simple and easily accepted by patients, however, its efficacy differs across patients with different tinnitus types.

We designed this study to explore which tinnitus patients are suitable for compound sound therapy and to predict the effectiveness of compound sound therapy for tinnitus patients. We analyzed 991 patients with subjective tinnitus and studied the clinical characteristics associated with compound sound therapy efficacy, such as the tinnitus type, tinnitus location, tinnitus sensation level, tinnitus frequency, and the tinnitus sound type. A predictive clinical nomogram was developed to evaluate the effectiveness of compound sound therapy based on clinical characteristics.

We analyzed the associations of demographic characteristics and tinnitus-related characteristics with the effects of sound therapy. In a study on the treatment of subjective tinnitus with repetitive transcranial magnetic stimulation (rTMS), Wang et al. recruited 289 patients with subjective tinnitus and provided each subject 1,000 1 Hz magnetic stimuli per day for 5 consecutive days per week for 2 weeks and found that age impacted on the efficacy of rTMS (25). In this study, however, we found that age had no effect on the efficacy of compound sound therapy. We believe that this discrepancy may be related to the fact that 52.5% of the subjects in this study had chronic tinnitus, and tinnitus persisted for more than 3 months. Most of the subjects in this study were 18–65 years old. This phenomenon can likely be attributed to several factors. First, the nature of the study was observational, with a focus on patients who presented with tinnitus at the outpatient Department of Otolaryngology. Second, individuals aged 18–65 who experience tinnitus may face heightened daily pressures, including work, study, and personal life stressors, which could contribute to the onset and exacerbation of tinnitus symptoms. Additionally, psychological factors may play a role in the development and progression of tinnitus in this age group. Furthermore, individuals in this demographic group may possess greater financial resources and a stronger motivation to seek treatment for their tinnitus, potentially leading to a higher rate of hospital visits among this population.

Frank et al. recruited 194 tinnitus patients, of whom 130 had bilateral tinnitus patients, 27 had right tinnitus, and 37 had left tinnitus. All patients received rTMS 2,000 times a day for 10 days. The researchers found that patients with left and bilateral tinnitus experienced a significant reduction in tinnitus severity after treatment, but those with right tinnitus did not (26). In this study, we found that patients with tinnitus on different sides had different sensitivities to compound sound therapy. However, at present, we do not know why this phenomenon occurs, and we will continue to explore this phenomenon in future studies. In this study we found that RIT may have an impact on the efficacy of compound sound therapy. Feldmann et al. reported that tinnitus disappeared briefly after sound stimulation (27, 28). The above phenomenon is called residual inhibition and occurs in 60–80% of people with tinnitus (29). Galazyuk et al. suggested that residual inhibition results from a reduction in the spontaneous discharge of central auditory neurons caused by sound stimulation (30). It is hypothesized that patients with a completely positive result on the RIT may exhibit increased sensitivity to sound therapy. Additionally, compound sound therapy has been shown to effectively suppress the self-discharge of central auditory neurons.

We found that patients with different tinnitus frequencies had different sensitivities to compound sound therapy. We searched the relevant literature and did not find relevant studies on this phenomenon. In the future, we will design relevant experiments to explore the relevant mechanisms of this phenomenon. In this study, the effectiveness of compound sound therapy was influenced by the FBT. The primary purpose of the FBT is to identify the pitch-to-loudness relationship characteristics of tinnitus under specific sound stimulation, validate the precision and controllability of the tinnitus frequency, and establish a foundation for diagnosis. The FBT involves the continuous application of sound stimulation at 1 dB increments above the tinnitus loudness threshold to assess changes in tinnitus perception, thereby providing insights into the patient’s responsiveness to compound sound therapy.

Predictive models are increasingly used in clinical decision-making and can provide personalized or precise medical guidance to patients (31). Ramakers et al. designed a cross-sectional retrospective study of 87 patients with bifocal severe hearing loss and tinnitus who received a unilateral cochlear implant. Tinnitus recovery was predicted by age, sex, the duration of deafness, preoperative hearing performance, duration, severity and location of tinnitus, the duration of follow-up, and the surgical approach. The researchers constructed a model to predict tinnitus recovery after cochlear implantation using variables such as a low preoperative consonant-vowel-consonant score, unilateral tinnitus, and the threshold of hearing deterioration in the operative ear at 250 Hz (32). Niemann et al. retrospectively analyzed a cohort of 4,117 tinnitus patients, analyzed 185 clinically relevant features, and constructed a prediction model comprising 6 clinically relevant features to help select appropriate treatment strategies for patients with chronic tinnitus complicated with or without major depression. Most previous studies have focused on predicting the occurrence of tinnitus, its impact on life and the efficacy of some treatment methods; few have focused on predicting the efficacy of compound sound therapy (33).

In this study, we constructed a prediction model for the efficacy of compound sound therapy on tinnitus, selected 13 variables, and used multivariate binary logistic regression. We found that FBT, RIT and the tinnitus frequency were all independent risk factors for evaluating the effectiveness of compound sound therapy for tinnitus. The AUC of the model constructed in this study was 0.766 (95% CI = 0.725–0.807), indicating a certain predictive ability. The correction curve also shows that the predicted results are in good agreement with the actual results. Through internal verification, we found that the AUC of the constructed model was 0.731 (95% CI = 0.662–0.800), which indicated that the constructed model had certain reliability.

This study had several limitations. First, all the data were retrospective, and numerous confounding factors were present. Second, the prediction model constructed in this study is based on data from a single medical institution and is not supported by data from other medical institutions. Therefore, external verification of the model in future studies is necessary to explore its feasibility. Finally, further determination of the reliability of the nomogram through prospective studies is necessary.

## Conclusion

After fully considering the demographic characteristics and tinnitus characteristics, we used FBT, RIT and the tinnitus frequency as three predictors to establish a multivariate logistic regression model and a nomogram for predicting the efficacy of compound sound therapy. The model can predict the prognosis of tinnitus patients receiving compound sound therapy and help otolaryngologists make the best clinical decisions regarding tinnitus treatment.

## Data Availability

The raw data supporting the conclusions of this article will be made available by the authors, without undue reservation.

## Footnotes

^1^ http://www.r-project.org/
